# Supplementary material for: In situ nanoscale imaging of moiré superlattices in twisted van der Waals heterostructures
Source: Nat Commun. 2020 Aug 21;11:4209. doi: 10.1038/s41467-020-18109-0 (PMC7442634; doi:10.1038/s41467-020-18109-0)
Supplement: Supplementary file 1 — Supplementary Information [file 41467_2020_18109_MOESM1_ESM.pdf]

In-situ nanoscale imaging of moiré superlattices in twisted van der Waals heterostructures

Yue Luo[1,2], **Rebecca Engelke**[2], **Marios Mattheakis**[3], **Michele Tamagnone**[3], **Stephen Carr**[2], **Kenji Watanabe**[4], **Takashi Taniguchi**[4], **Efthimios Kaxiras**[2,3], **Philip Kim**[2], **William L. Wilson**[1,*]

[1]Center for Nanoscale Systems, Harvard University, Cambridge, MA, 02138, USA

[2]Department of Physics, Harvard University, Cambridge, MA, 02138, USA

[3]John A. Paulson School of Engineering and Applied Science, Harvard University, Cambridge, MA, 02138, USA

[4]National Institute for Materials Science, Namiki 1-1, Ibaraki 305-0044, Japan

[*]e-mail: wwilson@cns.fas.harvard.edu

Supplementary Note 1: Graphene transport quality measurement

Two-probe resistance of one of the graphene stacks was measured at 10 K (Supplementary Fig. 1). The intrinsic doping at 0V is observed to be 1.4×10^{11} electrons per cm^2, a typical value for encapsulated graphene devices, implying no unusual doping from the substrate. The bottom h-BN was 20 nm thick and the top h-BN was 4 nm. After multiple steps of electron beam lithography there was polymer residue on top of the stack. Polymer residue on top of thin top h-BN explain the broad Dirac peak compared to standard encapsulated graphene devices. However, the clearly formed Dirac peak indicates that it is possible to do transport measurements in this system, even more promising with the possibility of using thicker top h-BN in the range of 8-16 nm.

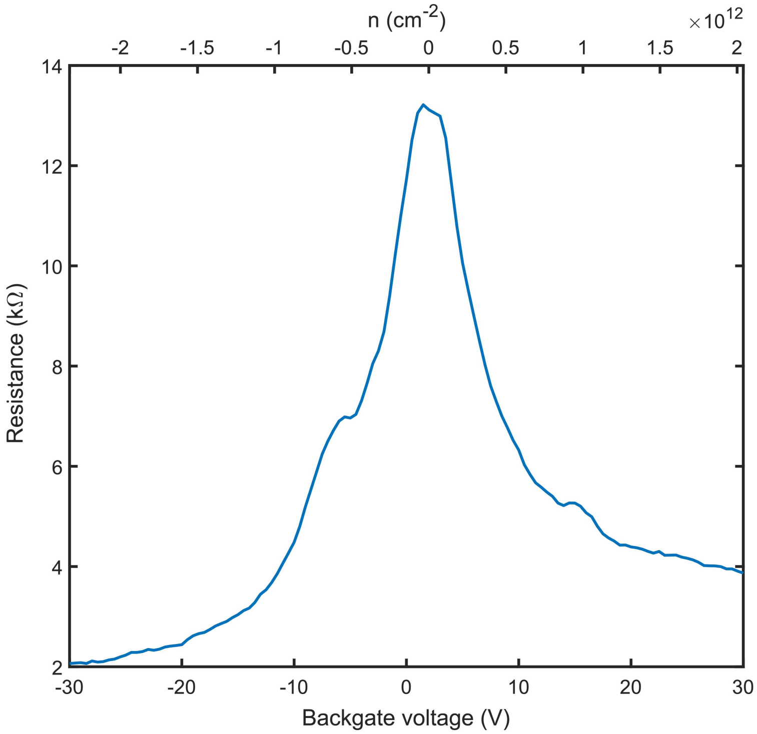

Supplementary Figure 1. Backgate voltage dependent resistance for the graphene encapsultated by h-BN. Data recorded at 10 K.

Supplementary Note 2: Soliton conductivity in bilayer graphene

To understand the local conductivity profile near the domain walls of small-angle twisted bilayer graphene (TBG), we employ a simplified tight-binding Hamiltonian for 1D soliton geometry. Each graphene layer has a nearest-neighbor bonding length of 1.42 Å and we consider only nearest-neighbor in-plane coupling of strength 2.7 eV. Longer range in-plane couplings would give a more accurate picture of graphene's low-energy electronic structure by capturing e.g. particle-hole asymmetry and trigonal warping. The interlayer coupling is captured using just the radially symmetric part, V_0, of an interlayer coupling functional obtained from a DFT study[1]:

$$V_0(\boldsymbol{r}) = \lambda_0 e^{-\xi_0 \bar{r}^2} \cos(\kappa_0 \bar{r}) \tag{1}$$

with $\lambda_0 = 0.3155$ eV, $\xi = 1.7543$, $\kappa_0 = 2.001$, and $\bar{r} \equiv r/a$ for r the in-plane distance between orbitals and $a = 2.46$ Å the lattice parameter of graphene. As this function depends on only r, the interlayer distance between layers plays no role in our simple model.

The soliton between an AB and BA stacking domain is known to be a shear deformation, with the atomic displacements parallel to the soliton line. In TBG, the soliton lines occur perpendicular to the nearest-neighbor bonding directions of the graphene unit cell. We define the graphene unit cell with primitive vectors $\boldsymbol{a}_1 = a(1,0)$ and $\boldsymbol{a}_2 = \frac{a}{2}(1,\sqrt{3})$, again with $a = 2.46$ Å. To create a periodic 1D soliton that agrees with those in TBG, the atomic displacements must therefore be in the $\hat{y}$ direction. This is most easily implemented by constructing a rectangular shaped supercell, with the cell defined by vectors $ma\hat{x}$ and $a\sqrt{3}\hat{y}$. This is effectively an $m \times 2$ supercell of graphene, but with an updated boundary condition to ensure uniformity in the $\hat{y}$ direction (up to a 60° rotation). To keep our system periodic, we must introduce two solitons: one transforms the bilayer from an AB to BA stacking, and then the second moves it back. The two layers begin in AB alignment with the top layer at a relative displacement of $(\boldsymbol{a}_1 + \boldsymbol{a}_2)/3$ to the bottom. We will perform a shear deformation by adding and then removing an additional $\boldsymbol{u}_0 = (0, -a/\sqrt{3})$ displacement (the nearest-neighbor bonding distance, in the $-\hat{y}$ direction), This additional displacement is evenly split between the two layers.

For the first soliton, centered at x_0 with a width w, this additional atomic displacement $\boldsymbol{u}$ is defined by a linear ramping function $s(x)$:

$$\boldsymbol{u}(\boldsymbol{r}) = \boldsymbol{u}_0 s(x), \quad s(x) = \begin{cases} 0 & x \leq x_0 - w/2 \\ \frac{x-(x_0-w/2)}{w} & x_0 - w/2 \leq x \leq x_0 + w/2 \\ 1 & x_0 + w/2 \leq x \end{cases} \tag{2}$$

with the second soliton "undoing" the transformation with an inverse function. Both soliton widths are chosen to be $w = 10$ nm, and have centers defined by $x_0 = ma/4$ and $x_0 = 3ma/4$. From this geometry, the electronic tight-binding hamiltonian is constructed, and a uniform $\boldsymbol{k}$-sampling of the rectangular supercell's Brillouin zone is performed, providing the eigenpairs $\{\varepsilon_{n\boldsymbol{k}}, \psi_{n\boldsymbol{k}}\}$ for band n.

A formula for optical conductivity that depends on only these eigenpairs can be derived from linear-response theory[2,3]:

$$\sigma(\omega)_{\mu\nu} = \frac{ig_s\hbar}{4\pi^2} \int d^2\boldsymbol{k} \sum_{m,n} \frac{f_n - f_m}{\varepsilon_m - \varepsilon_n} \frac{M_\mu^* M_\nu}{\hbar(\omega + i\eta) - (\varepsilon_m - \varepsilon_n)} \tag{3}$$

where the $g_s = 2$ accounts for the spin degeneracy in graphene, the $\boldsymbol{k}$ index is assumed for each band variable, the f_j are the Fermi occupation numbers for ε_j, and

$$M_\mu = \langle \psi_{m\boldsymbol{k}} | \boldsymbol{j}_\mu | \psi_{n\boldsymbol{k}} \rangle \tag{4}$$

for j_μ the current operator in the $\hat{\mu}$ direction. In our discrete tight-binding model, the current operator can be represented by way of a commutator, $\boldsymbol{j} = \frac{e}{i\hbar}[H, \boldsymbol{r}]$, which means the matrix elements M_i depend only on H and differences in position, $\Delta\boldsymbol{r}$, between orbitals. The variable η is a dampening rate defined by the electronic relaxation time, but here we use it as a tunable parameter to smooth the response function due to the discrete nature of our eigenvalue sampling in energy.

But we are more interested in a *local* conductivity. We can consider the local contributions to σ by defining the total conductivity over an area A in terms of local contributions, e.g.

$$\sigma = \frac{1}{A}\int_A d\boldsymbol{r}\sigma_{\boldsymbol{r}}. \tag{5}$$

Inspection of Eq. 3 reveals that a natural definition of $\sigma_{\boldsymbol{r}}$ is possible by expanding the eigenfunctions into local projections, $|\psi_{n\boldsymbol{k}}\rangle = \sum_A |\psi_{n\boldsymbol{k}}(\boldsymbol{r})\rangle$. Since we are dealing with a discrete tight-binding model, it will be more natural to represent the integral over A with a discrete sum over finite-sized regions, A_i. We define P_i as the projection operator onto atomic orbitals in the region A_i, e.g. $P_i|j\rangle = 1$ if orbital j is in A_i, and 0 otherwise. Then our local $\boldsymbol{j}$ matrix elements are:

$$M_\mu(i) = \langle\psi_{m\boldsymbol{k}}|\,\boldsymbol{j}_\mu P_i\,|\psi_{n\boldsymbol{k}}\rangle \tag{6}$$

This allows us to reexamine the conductivity formula in terms of the local contributions of the eigenvectors. By defining

$$\Sigma_{\mu\nu}(\omega,i,j) = \frac{ig_s\hbar}{4\pi^2}\int d^2\boldsymbol{k}\sum_{m,n}\frac{f_n - f_m}{\varepsilon_m - \varepsilon_n}\frac{M^*_\mu(j)M_\nu(i)}{\hbar(\omega + i\eta) - (\varepsilon_m - \varepsilon_n)} \tag{7}$$

one can obtain the local conductivity through

$$\sigma(\omega)_{\mu\nu} = \sum_{ij}\Sigma_{\mu\nu}(\omega,i,j) \equiv \frac{1}{A}\sum_i A_i\sigma_i \tag{8}$$

giving

$$\sigma_i(\omega) = \sum_j \frac{A}{A_i}\Sigma(\omega,i,j). \tag{9}$$

The results for the local conductivity (in units of $e^2/\hbar$) are shown in Supplementary Fig. 2. A clear peak in the conductivity of the soliton regions (domain-walls, or DW) is visible near $\omega = 0.2$ eV, and is accompanied by a negative imaginary component. The conductivity at center of the AB shows a much smaller feature, possibly due to its proximity to the two 10 nm wide solitons in our 36.9 nm wide periodic geometry. In a system with near infinite AB domains, this feature should be absent. At $\omega = 0.22$ eV, the conductivity at the AB domain is roughly 0.5 $e^2/\hbar$, which is twice the conductivity of monolayer graphene. The solitons (DW) show a large enhancement of the conductivity in this frequency range, with a value of roughly 1.1 $e^2/\hbar$ at their center.

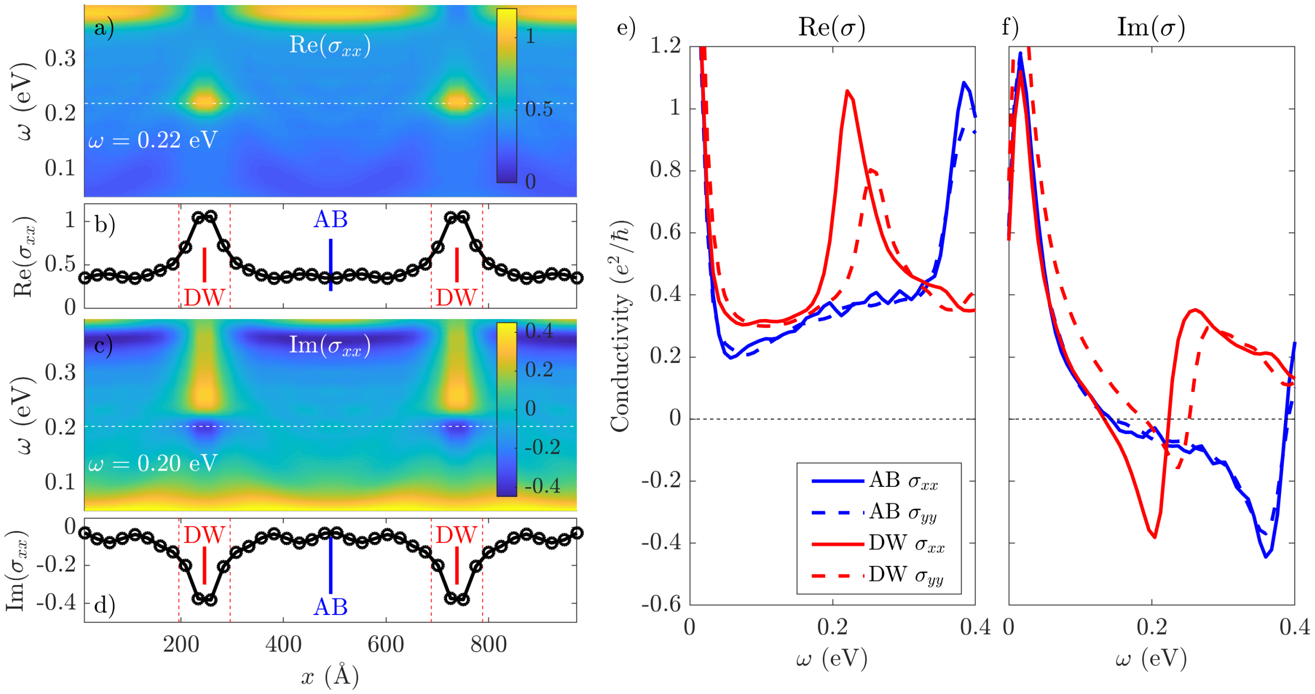

Supplementary Figure 2. Frequency dependence of the real and imaginary parts of the local conductivity in the bilayer graphene soliton model. a, Real part of the local conductivity, in units of $e^2/\hbar$, given by the color bar. The dashed white-line indicates the frequency where the cross-section is taken. **b**, Cross-section of **a**, with the domain-wall (DW) and AB stacking area indicated by the red and blue lines, respectively. **c, d**, Same as **(a, b)**, but for the imaginary part of the local conductivity. **e, f**, Spatial dependence of the real and imaginary parts of the local conductivity. The supercell is defined by $m = 400$ (984 Å in width) and a soliton width of 10 nm. The conductivity was calculated over 500 uniformly sampled $\boldsymbol{k}$ points, a Fermi energy of 50 meV (no vertical displacement field) to match the intrinsic doping of the experimental devices. The damping rate is taken to be $\eta = 0.01$, to ensure sufficient smoothness in the frequency-dependent curves.

Supplementary Note 3: Dispersion relation and nano-FTIR spectrum

In this note we perform a quantitative analysis of the dispersion relation and calculate the absorption spectrum of a heterostructure that consists of h-BN and TBG. We explore these features in the AB regime and along the domain wall (DW) of the Moire pattern (shown in Fig. 1b in the main text). The TBG is placed between two h-BN thin slabs of thickness $d \simeq 25$ nm and $\ell \simeq 4$ nm. We are interested in the frequency range $\omega = [1370\ \text{cm}^{-1}, 1650\ \text{cm}^{-1}]$ where optical phonon modes in h-BN are supported. Our TBG samples are designed with twisted angles with $\theta < 0.1^o$ where the length of the DWs is estimated about 140 nm (we use the rule for the length $\alpha/\sin(\theta)$, where $\alpha = 0.246$ nm). Our measurements are taken far away of the AA spot of TBG and we assume that 140 nm is a sufficient distance to avoid electronic contributions from the AA spots. Subsequently, the dispersion relation at DW and AB is assumed to be similar with the dispersion observed in bilayer graphene with a single DW, along and far away of the DW, respectively. Hence, in our theoretical modeling the TBG conductivity for the AB and DW is considered the same with the conductivity in at the AB and DW a bilayer graphene with a structural soliton. In addition, we neglect the thinner h-BN layer. More specifically, in our model we assume a heterostructure that consist of three regions along the z direction and infinitely extended along the $x-y$ plane. The three layers are considered as: air at $z > 0$ $(j = 0)$, h-BN at $0 > z > -d$ $(j = 1)$, and SiO_2 at $z < -d$ $(j = 2)$. The dielectric functions of the air is considered $\varepsilon_0 = 1$ and of the SiO_2 is $\varepsilon_2 = \varepsilon_s = 3.9$. For the h-BN we read an anisotropic and frequency dependent dielectric tensor with in- and out of plane components $(\varepsilon_x(\omega), \varepsilon_z(\omega))$, respectively, that follows the Lorentz model:

$$\varepsilon(\omega) = \varepsilon_\infty \left(1 - \frac{\omega_{\text{LO}}^2 - \omega_{\text{TO}}^2}{\omega^2 - \omega_{\text{TO}}^2 + i\omega\Gamma}\right). \tag{10}$$

The modeling parameters used are shown in Supplementary Table 1[4].

	ω_{TO} (cm^{-1})	ω_{LO}(cm^{-1})	Γ(cm^{-1})	ε_∞
ε_x (in-plane)	1366	1610	7	4.87
ε_z (out-plane)	760	825	3	2.95

Supplementary Table 1. Modeling parameters for the dielectric function of h-BN.

Supplementary Fig. 3 shows the h-BN dielectric function.

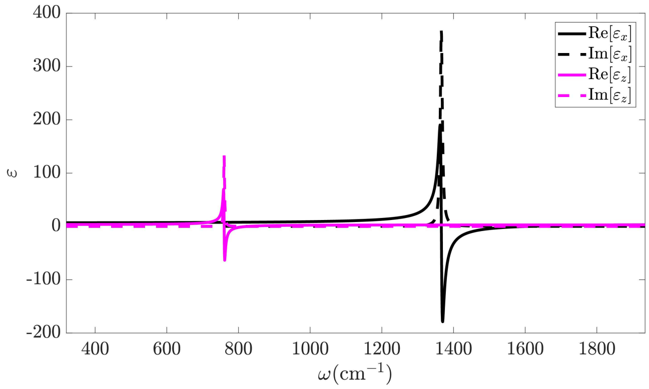

Supplementary Figure 3. The dielectric function of h-BN. The peaks indicate phonon resonances.

The TBG is located at $z = 0$ and is treated as a surface current $J_s = \sigma_{xx} E_x$, where σ_{xx} is the space and frequency de-

pendent $x-$component of the optical conductivity tensor for graphene bilayer; the spatial dependence appears due to the structural soliton formation. The complex conductivity is calculated by the tight-binding model described in Supplementary Note 2 and shown in Supplementary Fig. 2. The intra-band electronic transitions dominate at small frequencies and therefore, the imaginary part of the conductivity is positive. In this frequency range, transverse magnetic (TM) plasmons are supported and hybridize with optical phonons in h-BN[5,6]. On the other hand, in the frequencies that our experiments are performed the inter-band electronic transitions dominate the optical properties. This is a crucial point in this study since the imaginary part of the conductivity becomes negative and only transverse electric (TE) polarized plasmons are supported[7]. However, in s-SNOM experiments the TE polarized modes are very weak and can be neglected[5]. Hence, for the remaining TM polarization the negative $\mathrm{Im}(\sigma)$ makes the environment optical denser while the positive $\mathrm{Re}(\sigma)$ introduces dissipation. We observe in Fig. 2 that along the soliton line the dissipation is enhanced and the medium becomes optical denser affecting significantly the phonon modes. The phonon resonance becomes weak and, more specifically, along the DW the phonons become even weaker than in AB regime. Moreover, the presence of graphene bilayer weakly affects the dispersion relation and thus, the wavelength of the phonon polariton modes is slightly changed.

In order to understand qualitatively how the presence of TBG affects the phonon polariton propagating modes we employ Fresnel's equations to calculate the total complex reflectivity of the air/h-BN/TBG/SiO_2 heterostructure. We follow similar formulation used in Ref.[5]. We consider TM polarized waves with the magnetic and electric fields $\mathbf{H}(x,z) = (0, H_y, 0)$ and $\mathbf{E}(x,z) = (E_x, 0, E_z)$, respectively; the fields are invariant along the y direction. Assuming electromagnetic waves that propagate along the x-direction we get for the in-plane fields $H_y(x,z) = h(z)e^{iqx}$ and $E_x(x,z) = e_x(z)e^{iqx}$, where h and e_x are the associate field amplitude functions and q is the wavenumber along the x-direction. In each of the four layers of the heterostructure, the amplitude field for the magnetic field can be written as the sum of two counter-propagating waves as:

$$h^j(z) = A_j e^{-i\kappa_j z} + B_j e^{i\kappa_j z}, \tag{11}$$

where the subscript j denotes the layer and κ_j is the the out-plane wavenumber given by

$$\kappa_j = \sqrt{\frac{\varepsilon_x^j}{\varepsilon_z^j}} \sqrt{\varepsilon_z^j k_0^2 - q^2}. \tag{12}$$

Accordingly, the amplitude function of the electric field is written:

$$e_x^j(z) = -\frac{\eta_0 \kappa_j}{\varepsilon_x^j k_0} \left(A_j e^{-i\kappa_j z} - B_j e^{i\kappa_j z} \right), \tag{13}$$

where $k_0 = \omega/c$ and $\eta_0 = \sqrt{\mu_0/\varepsilon_0}$ are the wavenumber and the impedance in free space, respectively. Similarly to Ref.[4] the reflection coefficients r_{ij} at the i/j interface are defined as

$$r_{ij} = \frac{Q_j - Q_i + S_{ij}}{Q_j + Q_i + S_{ij}}, \tag{14}$$

where

$$Q_j = \frac{\varepsilon_x^j}{k_j}, \qquad (\varepsilon_x^j \text{ is the in-plane permittivity of the } j^{\text{th}} \text{ layer}), \tag{15}$$

$$S_{ij} = \frac{\eta_0}{k_0}\sigma_{ij}, \qquad (\sigma_{ij} \text{ is the conductivity along the } i/j \text{ interface}). \tag{16}$$

Since the TBG is placed at the interface between air and h-BN, the only nonzero conductivity terms are the $\sigma_{01} = \sigma_{10} = \sigma_{xx}$. The total complex reflectivity is calculated as:

$$r_p = \frac{r_{01} + r_{12}(1 - r_{01} - r_{10})e^{2ik_1 d}}{1 - r_{10} r_{12} e^{2ik_1 d}}. \tag{17}$$

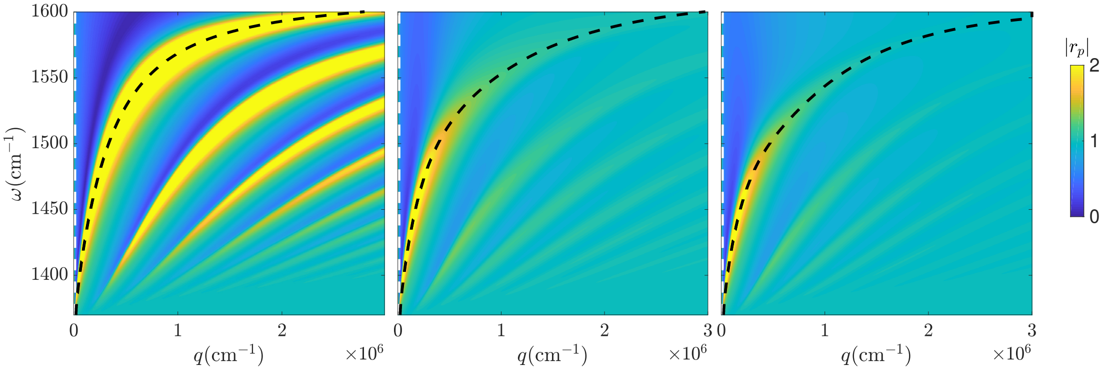

Supplementary Figure 4. The false-color indicates the absolute value of the reflection coefficient r_p which determines the dispersion relation. The maxima of $|r_p|$ denote phonon polariton modes in the h-BN/TBG heterostructure. Left: The conductivity of TBG is set to zero. Middle: Dispersion relation in AB regime of TBG. Right: Dispersion relation along the TBG DW. In each image, the black dashed lines show the first phonon polariton propagating mode while the white dashed lines are the far field modes corresponding to the light line.

In the absence of any dissipation, i.e. for an ideal system, the dispersion relation $q(\omega)$ can be determined by the poles of Eq. 17 where the r_p diverges. However, our heterostrucure includes dissipation, hence r_p never diverges at any real ω and q[4,5]. Here, the dispersion relation is visualized from the absolute value of r_p and the propagating phonon modes are graphically determined from the maxima of $|r_p|$. In Supplementary Fig. 4 we show the dispersion relation (false color) and outline the propagating modes (dashed lines). In particular, the false color shows the calculated $|r_p|$, the white dashed lines indicate the light lines (far field propagating modes), and the dashed black lines represent phonon modes (near field propagating modes). We present three different cases: In the left and for sake of comparison, we show the dispersion of a system without considering TBG ($\sigma_{xx} = 0$). The middle and right images show the dispersion of the heterostructure including the TBG. Specifically, the middle plot represents the AB regime ($\sigma_{xx} = \sigma_{\mathrm{AB}}$), whereas, the right image is the dispersion along the DW ($\sigma_{xx} = \sigma_{\mathrm{DW}}$). We observe that the phonon modes becomes very weak due to the presence of TBG. Essentially, the phonon modes disappear quickly as the wavenumber (or the frequency) increases. It is worth noting that along the DW the phonon amplitude is weaker than that in AB regime. This is expected due to the steep increase of the dissipation along the DW for $\omega > 1400\ \mathrm{cm}^{-1}$.

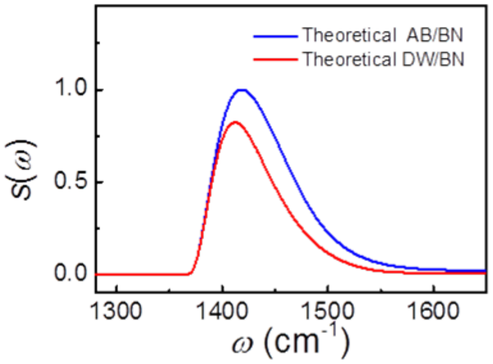

Supplementary Figure 5. Near-field spectra calculated for AB/BA domain (blue solid line) and along the domain wall (red solid line).

The wavenumber of the mode in AB regime (dashed black line in the middle graph of Supplementary Fig. 4) is used to calculate the phonon wavelength shown with dashed line in Fig. 2g. A qualitative correspondence between the s-SNOM near field signal (Supplementary Fig. 5)) can be performed by calculating the maxima of

the $|r_p|^2$ along the modes[8] (black dashed lines in middle and right images of Supplementary Fig. 4). These results are presented in Supplementary Fig. 5), where indeed we observe that along the DW the scattering field is weaker than the field along the AB regime. It is known that this approach introduce a blue shift of 40cm^{-1}[9,10]. However, Fresnel's equations capture the overall behavior and thus, this model is sufficient to explore and understand the underlying phenomenon. A more accurate description requires modeling that includes the tip-sample coupling. These simulations can be perform with numerical methods such as FDTD or by using approaches as the rod model that is introduced in Ref. 10.

Supplementary Note 4: FDTD simulations

Simulation setup

FDTD simulations are performed in Lumerical FDTD Solutions. The gaussian conductivity profile is implemented step-wise with steps of 2 nm (see below for more details). For the simulation of the profile of polaritons, the mesh size was set to 1 nm, and the tip was emulated with a vertically polarized Hertzian dipole placed at the interface between the top h-BN and air combined with electric field monitors at different heights just above the Hertzian dipole. The field emitted by the dipole is coupled to polaritons, which are reflected by any nearby discontinuity, and the reflection is measurable using field monitors in the simulations. The simulation is repeated for each tip position, and the real part of the z component of the electric field on the monitors is recorded for all wavelengths and used to generate the dispersion maps as function of wavelength and tip position.

FDTD Simulations shown in Fig. 2c are performed with a different setup designed to emulate the influence of the SNOM tip on the observed local spectrum of the sample.The simulation region has a size of $4\mu m \times 4\mu m$ and PML (perfectly matched layer) boundary conditions. The tip is simulated as a platinum pyramid suspended above the heterostructure. The tip is approximated with base of 20 nm, opening angle of 12 degrees, height 2.4 microns. A Broadband Fixed Angle Source Technique (BFAST) plane wave is incident on the tip and the sample and propagates at an angle of 60 degrees from the normal, and emulates the light incident from the spectrum. The power absorbed by the sample is then calculate placing a power field monitor between the tip and the sample. Simulations shows a clear red-shift of the spectrum due to the perturbation of the fields by the tip. A similar effect was also observed in literature for h-BN nanoresonators.

Constitutive equations of the materials

h-BN is modelled with a Lorentz model (Eq. 10) while TBG is modelled with 2D rectangles (boundary conditions). The TBG conductivity model is defined as follows. Away from the domain walls, the used conductivity is the one computed for the AB TBG case. The domain wall is computed using a spatially varying gaussian conductivity profile defined so that the conductivity is the one computed for the domain wall at the center of the gaussian, σ_{DW}, and it gradually averages to the one for the AB TBG case, AB. This is done using the formula:

$$\sigma_{\mathrm{TOT}} = p\sigma_{\mathrm{DW}} + (1-p)\sigma_{\mathrm{AB}} \tag{18}$$

where p is a normalized gaussian with standard deviation of 8 nm.

Background removal

As simulated, the dispersion maps have a significant background since the monitor captures part of the field emitted by the dipole and its image reflected by the sample. The background can be simulated at a location far from any discontinuity and subtracted frequency-by-frequency from the map to remove the background. The simulation can be repeated using the edge of the sample as a discontinuity, revealing a different reflection phase. Supplementary Fig. 6 shows an example of background removal.

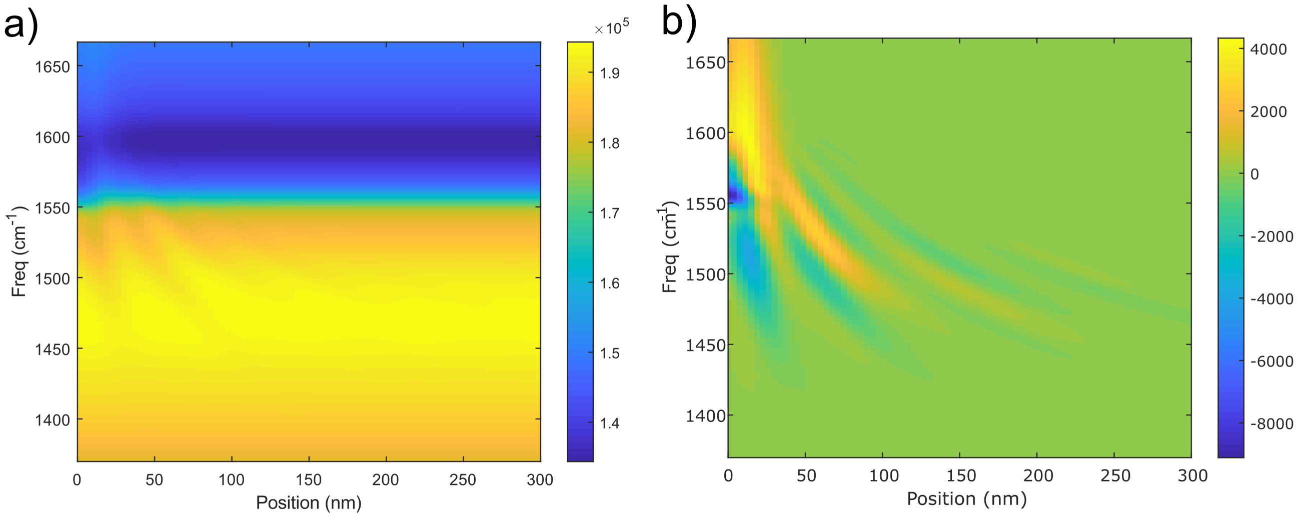

Supplementary Figure 6. **Removal of background in FDTD simulations.** **a**, Raw simulation results. **b**, Simulation results after subtracting the background

Higher order harmonic demodulation

Demodulation scheme is commonly used in the experiment and simulation. It will effectively reduce the far-field background. However, in the FDTD simulation, we have the monitor very close to the tip and sample and simulate the near-field effect without far-field scattering. Therefore the simulated spectrum is almost same regardless of the position where it is measured. As shown in Supplementary Fig.7, we performed additional simulations using the demodulation scheme to reconstruct the optical harmonics of the system. The results are very similar to the Fig.2c of the manuscript validating our assumption, especially at the resonance. When sampled right at the tip position in the simulation setup a demodulation scheme will not provide much benefit.

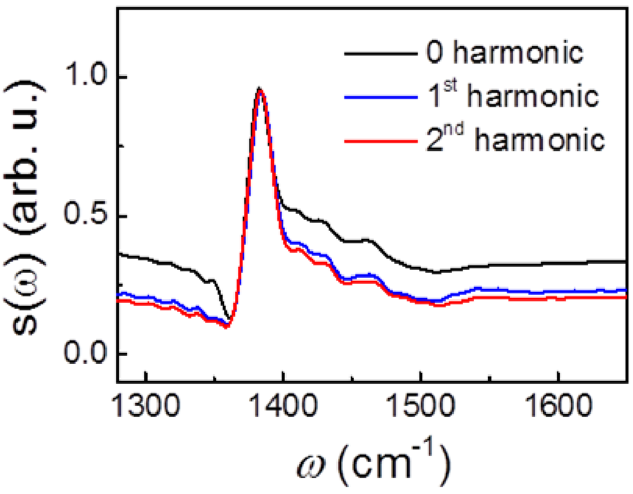

Supplementary Figure 7. **Higher order harmonic demodulation.** Near-field amplitude signal $s(\omega)$ simulated with a z-position demodulation scheme.

Post-processing of the polariton field profile

The electric field profile is obtained from the simulated maps taking a single frequency and plotting as a function of the tip position. Because the simulation considers an infinitely small point as the source, an additional smoothing is performed on the profile to emulate the tip size. Best fits are obtained using a window average with size 10 nm, which is in good agreement with the resolution of the tip.

Ideal FDTD simulations and reflection phase theory

The phase of the reflection coefficient due to the presence of the domain wall is not a trivial quantity, and instead it depends on multiple parameters of the studied physical system. The origin of this phase, however, can be explained in the context of Maxwell's equations, and therefore can be captured by an FDTD simulation. For better clarity and simplicity, we consider here a simplified and ideal model of the polariton propagating on the van der Waals heterostructure and incident on the DW discontinuity.

This formalism is valid when the polariton wavelength is significantly larger than the van der Waals total thickness and models the entire stack as a 2D conductivity σ that is the sum of the conductivity of each layer. Next, both FDTD simulations or analytical models based on the transmission line (TL) formalism can be used to predict the propagation of polaritons and the reflection on loads of different types[11].

According to the TL formalism, in the high confinement limit the polariton propagating across the heterostructure of arbitrary width W can be modelled as a transmission line with characteristic impedance Z_0 equal to[11]:

$$Z_0 = \frac{1}{2W\mathrm{Im}(\sigma)k_0 n_{\mathrm{eff}}} \tag{19}$$

where k_0 is the free space wavelength and n_{eff} is the polarition effective index. The high confinement limit is valid for $n_{\mathrm{eff}} \gg 1$ which is the case here. Under that limit, the characteristic impedance presented in Ref. 11 simplifies to the expression above, and is valid regardless of the specific origin of the conductivity (i.e. can model both plasmon and phonon polaritons or a combination of the two as in this case).

The TL model is explained as a combination of a capacitive shunt term and an inductive series term. The capacitive term models the electric field associated to the polariton, and the inductive represents the stack conductivity. The inductive part is given by the inductance per unit length, which reads:

$$\ell = \frac{n_{\mathrm{eff}} k_0 Z_0}{\omega} = \frac{1}{2W\omega\mathrm{Im}(\sigma)} \tag{20}$$

This equation implies that any variation in the conductivity σ can always be modelled as a series element in the transmission line. This is equivalent to condition (ii) as defined in Ref. 11. This is the case also for complex conductivities since all functions in the model are analytic functions, and hence can be immediately generalized to the complex case. The value of the series element is the difference of the nominal conductivity σ_{AB} and the actual conductivity at the discontinuity σ_{DW}. The reflection coefficient of a series element according to the TL formalism and assuming that the discontinuity acts as a small local perturbation of the conductivity, is:

$$\Gamma \propto (\sigma_{\mathrm{DW}} - \sigma_{\mathrm{AB}})L \tag{21}$$

where Γ is the complex reflection coefficient and L is the effective width of the discontinuity (here approximately 10 nm). As expected, if the complex conductivities σ_{DW} and σ_{AB} were the same, then the reflection coefficient would vanish, since the discontinuity would not actually exist. Because the previous equation holds for complex values, the phase of the reflection coefficient is determined by the phase of the difference of the conductivities.

To verify this, we performed ideal FDTD simulations creating different types of arbitrary discontinuities. Supplementary Fig. 8 shows the predicted standing waves for each type of discontinuity. Increasing or decreasing the imaginary part of the conductivity creates reflection phases at about 180 degrees from each other as expected. If a real part (that is optical losses) is added without changing the imaginary part, then the phase difference is about 90 degrees, confirming the model.

Due to the dependence of the reflection coefficient on the difference of the conductivity, the reflection phase can vary across a wide range, including the experimentally observed phase shift and according to the measurement wavelength.

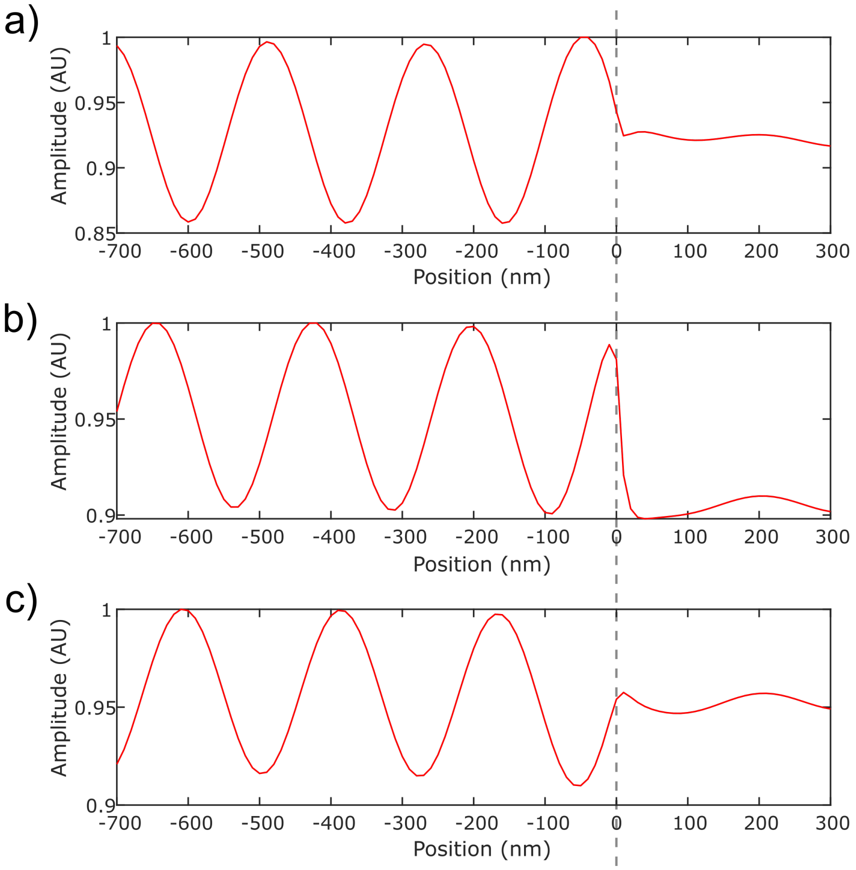

Supplementary Figure 8. FDTD Simulation of standing waves for different types of discontinuities. These simulations have been obtained with an ideal model of the polariton propagation. The discontinuity is placed at the origin of the position axis and the wave is travelling toward the right. **a**, Discontinuity having lower imaginary conductivity with respect to the rest of the stack and no real component. **b**, Discontinuity having same imaginary conductivity with respect to the rest of the stack and an additional real component. **c**, Discontinuity having higher imaginary conductivity with respect to the rest of the stack and no real component

Supplementary Note 5: Nano-imaging moiré superlattices with thick top h-BN

Since we directly use phonon-polariton in h-BN to image the moiré superlattices in TBG, we can have relatively thick h-BN on top to encapsulate the graphene layers without compromising the imaging contrast of the moiré pattern. As shown in Supplementary Fig. 9a, we test on the thick top h-BN region where the thickness is measured to be $\sim$ 16 nm. We can clearly map the moiré superlattices with the near-field amplitude at $\omega = 1550cm^{-1}$ (Supplementary Fig. 9b).

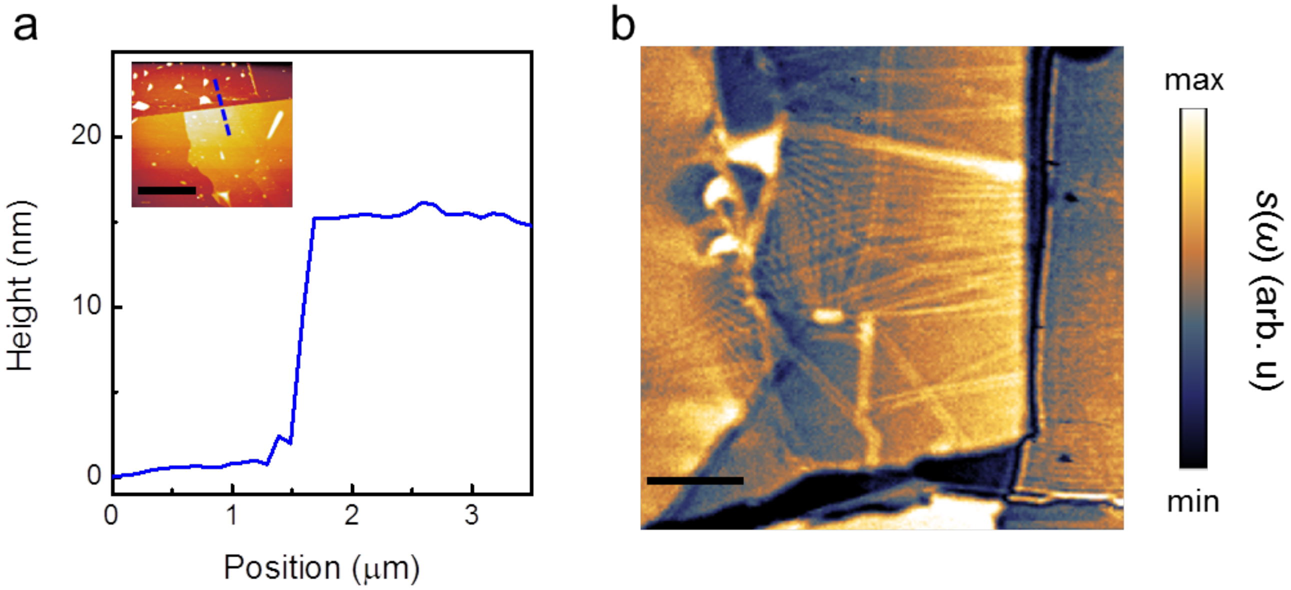

Supplementary Figure 9. Topography and near-field imaging with thick top h-BN. a, AFM profile of the sample region with 16 nm top h-BN. Inset: AFM height mapping of the sample. Blue dashed line indicates the profile location. Scale bar: $5\mu m$. **b**, Near-field images of the normalized near-field scattering amplitude $s(\omega)$ revealing the domain walls in TBG with 16 nm h-BN taken at $\omega = 1550cm^{-1}$. Scale bar: $1\mu m$

References

1. Fang, S. & Kaxiras, E. Electronic structure theory of weakly interacting bilayers. *Phys. Rev. B* **93**, 235153 (2016).
2. Jiang, B.-Y. *et al.* Plasmon reflections by topological electronic boundaries in bilayer graphene. *Nano Lett.* **17**, 7080–7085 (2017).
3. Massatt, D., Carr, S. & Luskin, M. Efficient computation of kubo conductivity for incommensurate 2d heterostructures. *The Eur. Phys. J. B* **93**, 60 (2020).
4. Dai, S. *et al.* Tunable phonon polaritons in atomically thin van der waals crystals of boron nitride. *Science* **343**, 1125–1129 (2014).
5. Dai, S. *et al.* Graphene on hexagonal boron nitride as a tunable hyperbolic metamaterial. *Nat. nanotechnology* **10 8**, 682–6 (2015).
6. Brar, V. W. *et al.* Hybrid surface-phonon-plasmon polariton modes in graphene/monolayer h-bn heterostructures. *Nano letters* **14 7**, 3876–80 (2014).
7. Mikhailov, S. A. & Ziegler, K. New electromagnetic mode in graphene. *Phys. Rev. Lett.* **99**, 016803 (2007).

8. Jiang, B.-Y. *et al.* Tunable plasmonic reflection by bound 1d electron states in a 2d dirac metal. *Phys. Rev. Lett.* **117**, 086801, DOI: 10.1103/PhysRevLett.117.086801 (2016).

9. Zhang, L. M. *et al.* Near-field spectroscopy of silicon dioxide thin films. *Phys. Rev. B* **85**, 075419 (2012).

10. McLeod, A. S. *et al.* Model for quantitative tip-enhanced spectroscopy and the extraction of nanoscale-resolved optical constants. *Phys. Rev. B* **90**, 085136 (2014).

11. Tamagnone, M. & Perruisseau-Carrier, J. Predicting input impedance and efficiency of graphene reconfigurable dipoles using a simple circuit model. *IEEE Antennas Wirel. Propag. Lett.* **13**, 313–316 (2014).
